# Epidemiology of viral hepatitis in the Republic of Congo: review

**DOI:** 10.1186/s13104-017-2951-8

**Published:** 2017-12-02

**Authors:** Laure Stella Ghoma Linguissi, Celine Nguefeu Nkenfou

**Affiliations:** 1 Institut National de Recherche en Sciences de la Santé, Brazzaville, République du Congo; 2 Chantal Biya International Reference Centre for Research on Prevention and Management on HIV and AIDS, Yaounde, Cameroon; 3 Higher Teachers Training College, University of Yaounde I, Yaounde, Cameroon

**Keywords:** Hepatitis B virus (HBV), Hepatitis C virus (HCV), Republic of Congo

## Abstract

**Objective:**

Considered an endemic zone, Republic of Congo has a high seroprevalence rate of hepatitis B and C virus. To know the extent of hepatitis infection as a public health problem, we reviewed published literature and other sources for reports of these viral infections in the country.

**Results:**

High seroprevalence of HBV and HCV carriage in blood donors were observed in studies confirming Congo’s place in the hyperendemic area of HBV and HCV infection. These prevalence were compared by Chi square test. We compared the prevalence of three studies conducted in 1996, 2015 and 2016. The statistical results were very significant. HBV genotype E was most prevalent. Very few studies were done on pregnant women. Difficulties in the care and management of patients were also noted because of the high cost of often unavailable treatments. Difficulties arise, however, when an attempt was made to implement the National Hepatitis Control Program. Despite studies conducted on hepatitis prevalence, health interventions are still needed to care and manage these patients and the need to implement the national hepatitis control is more pressing in the Congo.

## Introduction

Viral hepatitis is caused by five types of viruses (A, B, C, D and E) that are transmitted differently [1]. Type A and E are transmitted through food and water [2]. Infection with these viruses can lead to epidemic outbreaks of hepatitis in populations without safe drinking water and in poor sanitation conditions [3–5]. These two types of hepatitis (A and E) do not cause infection or chronic liver disease and do not require specific treatment [6, 7]. Types B and C are infections transmitted by blood during injection or medical interventions performed in poor sanitary conditions [8–11]. Hepatitis B virus is also transmitted sexually [12–14]. There is also transmission from mother to the child [15–18] which turns out to be the most important in the Congo, where pregnant women were found with significant proportions of hepatitis markers [19, 20].

Considered an endemic zone, Congo has a high seroprevalence rate of hepatitis C virus. A study conducted in 2014 among 17,351 blood donors, in the two major cities of the country revealed that the relative frequency was 4.1% in Brazzaville and 4.3% in Pointe-Noire [21]. At Brazzaville, the seroprevalence rate among 1363 blood donors was documented to be 9.9%. Hepatitis B infection remains also a public health problem in Congo [22].

To appreciate the extent of hepatitis infection as a public health problem in Republic of Congo, we reviewed published literature and other sources for reports of viral hepatitis in the country. The objective of this review is to report the overall prevalence of viral hepatitis in the Republic of Congo.

## Methods

### Main text

Although there is no comprehensive report on viral hepatitis in ROC, all studies focusing on hepatitis in the ROC have been considered. PubMed database was used to retrieve research articles about viral hepatitis in ROC. The search terms were: ((“hepatitis”[MeSH Terms] OR “hepatitis”[All Fields] OR “hepatitis a”[MeSH Terms] OR “hepatitis a”[All Fields]) AND (“congo”[MeSH Terms] OR “congo”[All Fields])) NOT (Democratic [All Fields] AND Republic [All Fields] AND (“congo”[MeSH Terms] OR “congo”[All Fields]).

### Criteria of inclusion

This review considered full articles and abstracts published in peer review journals between 1952 up to 2016 which have reported incidence and prevalence of viral hepatitis in Republic of Congo.

### Target population

Most studies on hepatitis in the Republic of Congo reported on blood donors. So our conclusion will be made based on blood donors.

### Strategy

Data were extracted and verified in full by one author (GLLS) on the following: country, year of publication, year of collection; study type, study design, biomarkers tested, study population, sample size, proportion male, age, HCV seroprevalence, PCR test based prevalence, genotype, subtypes.

### Analysis data

After collecting the related information, the data were transferred into the computer and analyzed by EpiInfo version 7.1.1. Data were analyzed using descriptive statistical methods, Chi square and correlation at a significant level of < .05.

## Results

We retrieved 16 research articles from the PubMed database. We excluded 7 articles from this review because their content did not mention any subtype. Table 1 summarizes the description of studies on hepatitis in the Republic of Congo.

**Table 1:** Description of selected studies

Study	Locality study	Study main objective	Type of population	Sample size	Infection prevalence n (%)
Atipo-Ibarra et al. [22]	Area: Pointe-Noire, Niari, Bouenza Design: Cross-sectional	To characterize the molecular profile of HIV in highly endemic areas of Congo	Donors of blood	1363	HBsAg (9.9%)
Angounda et al. [19]	Area: Pointe-Noire Design: Prospective	To detect the patients at high risk of hepatitis disease progression	Chronic HBV patients	111	HBeAg (15.3%) Anti-HBe (73%)
Angounda et al. [19]	Area: Pointe-Noire Design: Prospective	To determine the prevalence markers and factors associated with HBV infection in blood donors	Donors of blood	648	HBsAg 6.6% HBeAg 1.1% Anti-HBe 8.2% Anti-HBs 13.6% Anti-HBc 62.7%
Alidjinou et al. [48]	Area: Pointe-Noire Design: Prospective	To investigate HCV and HIV co-infection among asymptomatic and treatment-naïve blood donors in Pointe-Noire, Republic of Congo	Donors of blood	7785	366 (4.7%)
Atipo-Ibara. et al. [21]	Area: Pointe-Noire and Brazzaville Design: Coss-sectional	To identify the genotypes of the hepatitis C virus in Congo	Donors of blood	17,351	
Bossali et al. [81]	Area: Pointe-Noire Design: Cross-sectional, descriptive and analytical	To estimate the prevalence of co-infection of hepatitis B and C viruses with HIV among parous women in Pointe-Noire	New mothers	302	9.6% VHB 6% VHC
Taty-Taty et al. [80]	Area: Brazzaville Design: Cross-sectional, descriptive	To determine the prevalence of chronic carriage of HBsAg and anti-HBc in Brazzaville	General population	Undefined	AgHBs (7.3%) Anti-HBc (67.21%)
Bossali et al. [82]	Area: Brazzaville Design: Multicenter study-retrospective and descriptive	To define the place of infection by hepatitis B in the etiology of cirrhosis and hepatocellular carcinoma diagnosed in Pointe-Noire	Patients received for cirrhosis and hepatocellular carcinoma, who had undergone the search for HBsAg	3276	63% AgHBs
Makuwa et al. [83]	Area: Brazzaville Design: Longitudinal	To know the significant links between the presence of markers of hepatitis B and that of HIV infection	People attended by the Department of Infectious Diseases of the Hospital Center of Makélékélé	334	Ag HBs (8.5) Ag HBe (4.2) Anti-HBe (4.2) Anti HBc (93.6)
Dokekias et al. [49]	Area: Brazzaville Design: Prospective	To evaluate the seroprevalence of anti-HCV antibodies in poly transfused subjects	Multitransfused patients: and control cases	132 multitransfused patients and 120 control cases	AcHCV: 26 out of 132 (19.7%) and control cases 9 out of 120 (7.5%)
Cantaloube et al. [23]	Area: Brazzaville Design: Cross-sectional	To carry out among the Bantu and Pygmy populations was (i) to determine the seroprevalence and viremia of HCV	887 donors identified	50	5.6% as seropositive for HCV [subgroups: Ngala (~ 12%); Teke (3.6%); Kongo (5.6%); Pygmies (3.8%)]

### Localisation of studies

Most of the studies were conducted in Brazzaville and Pointe-Noire and one study was conducted in several localities: Niari, Bouenza, Lekoumou among ethnic groups (Bantu et Pygmy) [23].

### Hepatitis: a public health concern

In Congo, many people carry the virus B and/or C [19–22]. However, most of them are unaware of this, believing that the only chronic viral disease is HIV/AIDS [24, 25]. This situation is exacerbated by the lack of a response strategy that incorporates specific awareness-raising actions [26–28]; co-infection with hepatitis viruses and HIV/AIDS [29], the low or non-existence of immunization coverage against the B virus [30–33] or a lack of knowledge of HIV status, even among health workers [34, 35]. In addition, the difficulties in caring and management of patients were also noted because of the high cost of often unavailable treatments. Vaccines are available to prevent hepatitis B at a cost of 27,000 FCFA for adults and 9000 FCFA for the child for three doses [36], vaccination is the best way to get immunity to these viruses [37–40].

Effective drugs are available, but treatment remains inaccessible to many patients because of its high cost. In the Republic of Congo, hepatitis C treatment costs about one million FCFA per month and lasts at least 1 year. For hepatitis B, there is no treatment as such in the country. Treatment is now being done using antiretrovirals for HIV control that also act on hepatitis B [41, 42]. The screening costs is currently 30,000 FCFA and the full biological testing is of the order of 450,000–500,000 FCFA [36].

### Epidemiology of viral hepatitis

#### Hepatitis B

The high incidence of HBsAg carriage in blood donors was observed in the Pointe-Noire, Niari and Bouenza departments, confirming Congo’s place in the hyperendemic area of HBV infection. Atipo-Ibara et al. [22] in their study observed the frequence of HBsAg carriage in blood donors in few localities of the country: Pointe-Noire (10.8%), Nkayi (9.3%), Dolisie (8.9%), Madingou (5.9%) and Mouyondzi (5.2%) [22].

Angounda et al. [19] indicated a prevalence of HBeAg was 15.3 and 73% of patients were anti-HBe positive. In this study Angounda et al. detected the Chronic HBV among 111 patients at high risk of hepatitis disease progression. And the presence of HBeAg was high in chronic active hepatitis (CAH) (41.18%) and HBeAb positive was higher in the age group 39–45 year with a prevalence of 56.7% [43].

The prevalence of serological markers of hepatitis B was higher in the study of Angounda et al. among 648 blood donors in Brazzaville, which confirms the high risk of blood transfusion [44]. Over all, although the prevalence of hepatitis B was different from one study to another (see Table 2), this prevalence especially for HBsAg was above 6%, which is considered high.

**Table 2:** Summary of the prevalence of hepatitis B according to selected studies and biomarkers

Study	Sample size	HBsAg prevalence, n (%)*	Anti-HBe prevalence, n (%)^µ^	Anti-HBc prevalence, n (%)^#^
Atipo-Ibarra et al. [22] (1)	1363	9.9		
Angounda et al. [43] (2)	648	6.6	8.2	62.7
Makuwa et al. [83] (3)	334	8.5	4.2	93.6

*p* value: *p* (1) → (2) < 0.0192; *p* (1) → (3) < 0.5*; *p* (2) → (3) < 0.026^µ^; *p* (2) → (3) < 0.00000^#^; *p* (2) → (3) < 0.29*

#### Hepatitis C

Central Africa is considered as a high-prevalence region of anti-HCV antibodies, detected in about 2–20% of the population [45–47]. In ROC, HCV seroprevalence studies have been conducted among blood donors [23, 48, 49] or carried out among the Bantu and Pygmy populations. And HCV seroprevalence was lower in Pygmies (3.8%) [23]. The similarly study demonstrated a low prevalence of HCV infection among pygmies living in Cameroon [50].

In the previous study conducted in 2014 in Pointe-Noire, seroprevalence of anti-HCV antibodies in the blood donors has been reported to 4.7% [48].

The last study conducted among adults in one HIV clinic in the capital, Brazzaville in 2010, showed that the prevalence of HCV antibody was 5.7%. Studies have revealed that the majority of HCV positive patients were predominantly male [21, 51].

The key problem with this explanation is that generally, blood donors are male [52] and there are multiple restrictions for blood donation by women [53], which are linked either to breastfeeding [54, 55], menstruation [56] or pregnancy [57].

### Genotyping of hepatitis

#### Hepatitis B

In the single study on the genotyping of hepatitis B in the Republic of Congo, 82 samples out of 111 (73.9%) were genotyped in the HBV S region. Of these, 58 (70.7%) subjects were infected with HBV genotype E and 24 (29.3%) were infected with HBV genotype A. Previously in Cameroon, the two prevalent genotypes identified were 69.6% HBV genotype E and 30.4% HBV genotype A among the HIV-1 infected patients [58]. HBV genotype E was also the most prevalent genotype in Angola, found in 87.5%, followed by genotype A in 10% [59]. Genotypes E and A are the most prevalent among blood donors in areas of high endemicity [43].

#### Hepatitis C

In the study conducted by Cantaloube et al. in 2010, the genotyping was performed through amplification and sequencing of a 339 pb amplicon located from position 8370 to 8708 in the NS5b region and a 357-nucleotide sequence located from position 1029 to 1385 in the E1 region. The results of this study showed 21 strains belonged to characterized subtypes, that is 4c in 8 (25.8%), 4h in 2 (6.5%), 4k in 3 (9.7%), and 4r in 8 (25.8%) [23]. The most common subtypes were 4c and 4r. All these subtypes were identified in a study conducted in DRC [60].

Atipo-ibara et al. used only the NS5B region, they obtained sequencing of 17 samples, 16 genotypes 4 (G4) and 1 genotype 2 (G2) [22]. The distribution of genotype 4 subtypes in this article shows great genetic diversity and predominance of one subtype G4. The epidemic history of HCV subtype 4c and 4r slowed considerably [23]. Similarly genetic diversity has been observed in Kinshasa in DRC by Iles et al. in 2013 and 2015 [60–62]. Zeba et al. in 2014 in Burkina Faso observed that HCV genotype 4 was the least frequent among the blood donors [63].

### Risk factors for hepatitis B and C

Blood transfusion remains the most important mode of transmission of hepatitis C [64, 65]. In the Republic of Congo, Elira et al. in 2003 reported the seroprevalence of anti-HCV antibodies among 252 multitransfused patients to be 1.7% [49]. In one of their previous study they observed a prevalence of 6.7% of anti-HCV antibodies in blood donors [51]. Atipo Ibara et al. in 2013 on the other side indicated that blood transfusion was a risk factor in 143 donors out of a total of 1363 (10.5%) donors [22].

Thus, such as HIV/AIDS, hepatitis C is a major concern in blood transfusion services that requires mandatory pre-transfusion screening [49, 66]. ELISA is the most common routine test for hepatitis C screening [67–69]. The risk of false positive reactions that are due to cross-reactions with other viruses is very common [70, 71].

### Global response: implementation of the national program for the control of viral hepatitis B and C

A strategic plan was drawn for 2016–2021 and has to be implemented [72, 73]. It focuses on public awareness and prevention as well as access to safe, affordable and effective care and treatment [74, 75]. One of the goals of this strategy is to reduce the number of new cases of chronic hepatitis by 30% by 2020 and 90% by 2030 [76, 77] and to reduce mortality rates due to hepatitis B and C by 10% by 2020 and by 65% by 2030 [78].

So far in the Congo, a short term national program for the control of viral hepatitis B and C drawed since 2014 could not assure affordability of drugs and the care of patients remained very costly in the country [36, 79]. The national program will be responsible for regular supply of medicines biological tests necessary for the care and management of patients suffering from hepatitis. This program will play an important role in the collection of statistical data in the Republic of Congo.

Difficulties arise, however, when an attempt was made to implement the National Hepatitis Control Program. The few studies conducted in ROC showed that, there were the unmet needs for routine HBsAg screening and implementation of immunization against HBV as effective means to prevent hepatitis B [22]. New strategies must be developed to include routine screening of pregnant women and implementation of vaccination programs for newborns [43].

## Conclusion

Hepatitis is a major health problem that requires greater attention in Republic of Congo.

Although prevalence vary from one study to another, this prevalence was high, above 6% for HBsAg, one of the most reliable markers for hepatitis B infection. Also, genotype B was the most prevalent. This review reports finding of significant public health importance revealing a high frequency of co-infection with HBV/HCV in HIV infected individuals in Congo with risk factors for co-infection in the population. This review also stress that blood to be transfused should undergo systematic analyses in order to exclude viral infections including hepatitis.

## Limitations

In this study we summarized insights of hepatitis virus; pathology which is not well explored in our country. This review based finding of previous studies; data incompleteness and poor document retention system were limitation of this study.

## Abbreviation

HBV: Hepatitis B Virus
HCV: Hepatitis C Virus
ROC: Republic of Congo
